# Genetic Basis of Cry1F-Resistance in a Laboratory Selected Asian Corn Borer Strain and Its Cross-Resistance to Other *Bacillus thuringiensis* Toxins

**DOI:** 10.1371/journal.pone.0161189

**Published:** 2016-08-12

**Authors:** Yueqin Wang, Yidong Wang, Zhenying Wang, Alejandra Bravo, Mario Soberón, Kanglai He

**Affiliations:** 1 State Key Laboratory for Biology of Plant Diseases and Insect Pests, Institute of Plant Protection, Chinese Academy of Agricultural Sciences, Beijing, 100193, People’s Republic of China; 2 Instituto de Biotecnología, Universidad Nacional Autónoma de México, Apdo. Postal 510-3, Cuernavaca, 62250, Morelos, Mexico; Universidade Federal de Vicosa, BRAZIL

## Abstract

The Asian corn borer (ACB), *Ostrinia furnacalis* (Guenée) (Lepidoptera: Crambidae), is the most destructive insect pest of corn in China. Susceptibility to the Cry1F toxin derived from *Bacillus thuringiensis* has been demonstrated for ACB, suggesting the potential for Cry1F inclusion as part of an insect pest management program. Insects can develop resistance to Cry toxins, which threatens the development and use of Bt formulations and Bt crops in the field. To determine possible resistance mechanisms to Cry1F, a Cry1F-resistant colony of ACB (ACB-FR) that exhibited more than 1700-fold resistance was established through selection experiments after 49 generations of selection under laboratory conditions. The ACB-FR strain showed moderate cross-resistance to Cry1Ab and Cry1Ac of 22.8- and 26.9-fold, respectively, marginally cross-resistance to Cry1Ah (3.7-fold), and no cross-resistance to Cry1Ie (0.6-fold). The bioassay responses of progeny from reciprocal F_1_ crosses to different Cry1 toxin concentrations indicated that the resistance trait to Cry1Ab, Cry1Ac and Cry1F has autosomal inheritance with no maternal effect or sex linked. The effective dominance (*h*) of F_1_ offspring was calculated at different concentrations of Cry1F, showing that *h* decreased as concentration of Cry1F increased. Finally, the analysis of actual and expected mortality of the progeny from a backcross (F_1_ × resistant strain) indicated that the inheritance of the resistance to Cry1F in ACB-FR was due to more than one locus. The present study provides an understanding of the genetic basis of Cry1F resistance in ACB-FR and also shows that pyramiding Cry1F with Cry1Ah or Cry1Ie could be used as a strategy to delay the development of ACB resistance to Bt proteins.

## Introduction

The Asian corn borer (ACB), *Ostrinia furnacalis* (Guenée), ranks among the most damaging lepidopteran pest of corn throughout China, causing significant economic losses every year [1]. Therefore, ACB has been the target of several different management strategies. Owing to resistance to a wide range of synthetic chemical insecticides, transgenic crops producing insecticidal protein toxins (Cry toxins) from *Bacillus thuringiensis* (Bt) have provided an effective way to control lepidopteran pests with no known adverse effect to humans, beneficial insects, or other non-target organisms, and resulting in reduced use of broad-spectrum insecticides [2, 3]. Field trials assessing Bt corn expressing Cry1F, Cry1Ab, Cry1Ac, and Cry1Ie have been approved in China. Previous data demonstrate that Bt corn can offer season-long protection against ACB and other lepidopteran pests [4, 5].

Since first commercialized in 1996, adoption of Bt crops has steadily increased, reaching 80 million hectares in 2014 across 28 countries [6]. Bt crops represent an important alternative to conventional insecticides in terms of superior efficacy as well as environmental safety and economic benefit. However, widespread adoption and prolonged use of single trait products may promote the evolution of resistance among target pest species. Bt resistance has evolved in several species, including *Plutella xylostella* [7],*Ostrinia nubilalis* [8], *Spodoptera frugiperda* [9–11], *Busseola fusca* [12], *Trichoplusia ni* [13], *Pectinophora gossypiella* [14] and *Diabrotica virgifera virgifera* [15]. In addition, multiple selection experiments under laboratory conditions have shown the widespread potential for resistance evolution to Bt among insect pest species following prolonged exposure to Bt toxins [16]. In order to preserve the long-term utility of this technology, adoption of appropriate resistance management strategies is necessary. Among the theoretical strategies for resistance management, application of the high-dose/refuge approach and pyramiding of two or more toxins with different modes of action have been most widely cited [17, 18, 19]. With the high-dose/refuge approach, a high expression level of the insecticidal protein is intended to reduce the fitness of heterozygote progeny. A high dose must be combined with a non-Bt refuge in order to maintain a pool of susceptible homozygotes that would mate with rare homozygous resistant individuals, thus diluting the resistance gene in the offspring population [20]. The success of this strategy depends on a variety of factors, including pest movement, mating patterns, low initial resistance allele frequency and the mode of inheritance of resistance, which is assumed to be recessive [21, 22]. The pyramiding (mixture) of toxins with different modes of action is based on the premise that a species cannot easily evolve resistance to multiple toxins because it would require multiple simultaneous, independent mutations in the genes encoding their receptors [23]. Furthermore, the strategy of applying toxin mixtures or rotations of different toxins is more likely to succeed if the inheritance of resistance to each toxin is recessive [24, 25].

Knowledge of the genetic basis of resistance to Bt toxins is important for understanding, monitoring, and managing resistance. Our primary objective in the present study was to determine the mode of inheritance for Cry1F resistance in laboratory-selected ACB-FR, including evaluation of maternal effects, sex linkage, dominance, and the number of loci influencing resistance. Experiments utilized an artificially selected Cry1F-resistant strain (ACB-FR), derived from field-collected ACB and characterized to exhibit Cry1F resistance. Also, the current study was conducted to assess cross-resistance patterns among various Bt toxins in the Cry1F-resistant colony. The results of this research have direct implications for the management of resistance for prospective Cry1F-containing corn products.

## Materials and Methods

### Insect Strains

Two laboratory strains of ACB, a Bt susceptible strain (ACB-BtS) and a Cry1F-resistant strain (ACB-FR), were used for this study. The ACB-BtS originated from a field collection in Liaoning Province within the corn region of northeastern China, and reared on artificial diet [26] in the laboratory for 23 generations without exposure to Bt toxins. The ACB-FR strain originated from a population (88 pairs of female and male moths derived from 948 diapause larvae) was collected in autumn of 2010 from corn fields in Shaanxi Province (in the summer maize region of central China). No permit for collecting insect samples was required by authorities. Their offspring were established as a laboratory colony and maintained using artificial diet and rearing techniques described by Zhou et al [26]. The susceptibility of this colony to Cry1F was tested just before selection for Cry1F resistace (at generation 6^th^ reared under laboratory conditions) in 2011. The LC_50_ was 0.64 μg/g (toxin/diet). The colony was initially exposed throughout larval development to Cry1F incorporated into the diet (50 ng/g, toxin/diet) and was steadily selected with increasing concentrations of Cry1F added into the rearing diet for 17 generations and then maintained at 25 μg/g (toxin/diet) for 32 generations. Specifically, the concentrations initially was 0.05 μg/g (Cry1F toxin/diet), and was increased to 0.1 μg/g in the 2^nd^ generation, 0.2 μg/g in the 3^rd^–4^th^ generation, 1.6 μg/g in the 5^th^-7^th^ generation, 6 μg/g in the 8^th^-10^th^ generation, 12 μg/g in the 11^th^-13^th^ generation, 24 μg/g in the 14^th^-16^th^ generation, and 25μg/g in the 17^th^ generation. The resistance characteristics were tested at the 49^th^ generation. Larvae were reared in isolation and maintained at 27 ± 1°C, 70–80% relative humidity (RH) under a 16:8 h light:dark (L:D) photoperiod. Resulting pupae were transferred to mating cages. Egg masses were deposited onto waxed paper lining the top of the cage and gathered daily.

### Bt Toxins

Trypsin-activated Cry1Ab, Cry1Ac and Cry1F (98% pure protein), used for diet bioassay, were produced by Marianne P. Carey, Case Western Reserve University, USA. Cry1Ie expressed as a recombinant protein in *E*. *coli* was purified via NiNTA affinity chromatography and Superdex-75 size-exclusion chromatography. Cry1Ie was protoxin. Cry1Ah expressed in engineered *Bacillus thuringiensis* subsp. *kurstaki* strain *cry1Ah* (HD-73) was trypsin-activated toxin. These samples were analyzed by SDS-PAGE (8%), and the protein concentrations were determined (Universal Hood II, Bio-Rad, USA) using bovine serum albumin (BSA) as a standard. Both proteins (>85% pure protein) were provided by the Biotechnology Group of Institute of Plant Protection, Chinese Academy of Agricultural Sciences. Test solutions of toxins were freshly prepared in distilled water with sodium carbonate in 50 mM sodium carbonate buffer pH10.

### Diet Bioassay

The susceptibility of neonates to each Bt toxin was assessed by diet-incorporation bioassay. Experiments for each Bt toxin were conducted using the following procedure. Bt test solutions were serial diluted; dilutions were added to an agar-free semi-artificial diet to form a testing medium [27]. The freshly prepared diet was dispensed into each well of 48-well trays and allowed to solidify. Neonate ACB larvae (<12 h after hatching) were transferred, one per well, and maintained at 27 ± 1°C with a photoperiod of 16:8 h (L: D) and 80% RH. The number of survivors and the weight of larvae surviving per treatment were recorded 7 days following infestation. Because the mean mass of an ACB neonate is approximately 0.1 mg, a larva that had not grown beyond the first instar and weighed ≤ 0.1 mg was considered dead. In each experiment, bioassays were replicated three times and included six to thirteen different concentrations from 0.01–5 μg/g (toxin/diet) for ACB-BtS and 0.05–1000 μg/g (toxin/diet) for ACB-FR and crosses, plus a negative control (only water applied to the diet).

### Statistical Analysis

The susceptibility of different insect strains or genetic crosses to Bt toxins was analyzed by probit regression using PoloPlus (LeOra Software) to calculate LC_50_ with 95% fiducial limits (FL), slope with standard errors (Slope ± SE), chi-square (χ^2^) values and resistance ratios (RR) with 95% fiducial limits. Chi-square results and slope parameters were used to determine the reliability of the data. RR is based on the concentration of toxin killing 50% of larvae for the resistant strain relative to a susceptible strain. The data of F_1_ reciprocal crosses between ACB-FR and ACB-BtS were also analyzed with the equality and parallel tests using PoloPlus.

### Maternal Effects and Sex Linkage

The maternal influence and potential for sex-linkage of the resistance were determined from the slope and FL of LC_50_ values of the F_1_ progenies derived from the mass of reciprocal crosses between resistant and susceptible strains. LC_50_ values were considered significantly different if no overlap in 95% FL was observed. To generate virgin females and males for the reciprocal crosses, pupae were separated by gender, and enough selected females and control males were pooled together in mating cages, and vice versa. Mass crosses provided enough offspring for multiple toxin testing and backcross.

### Estimation of Degree of Dominance

The effective dominance (*h*) at specific concentrations was calculated as

$$\text{h}=\left(W_{12}-W_{22}\right)/\left(W_{11}-W_{22}\right)$$ (1)

where W_11_,W_12_ and W_22_ are the fitness of the homozygous resistant parent, heterozygous offspring and homozygous susceptible parent, respectively. The fitness of the susceptible parent and the heterozygous F_1_ was estimated from the survival rate of the larvae at a specific treatment concentration divided by the survival rate of the resistant parent at the same concentration. W_11_ was assumed to be 1 at any treatment concentration. *h* varies from 0 (completely recessive) to 1 (completely dominant), with 0.5 indicating codominance [28, 29].

### Number of Loci Influencing the Inheritance

The data obtained in survival bioassays using the backcross progeny produced by mass crossing of F_1_ adults obtained from reciprocal crosses to the selected strain using the same procedure for reciprocal crosses were examined to determine the number of loci influencing the inheritance.

The null hypothesis tested in the standard back-cross method is that resistance is controlled by one locus with two alleles, S (susceptible) and R (resistant). If so, the parental R strain is 100% RR and the F_1_ offspring are 100% RS. Further, the RS × RR backcross will produce 50% RS and 50% RR offspring. If the null hypothesis is true, then Y_X_, the expected mortality in the RS × RR backcross offspring at concentration x, is calculated as

$$Y_{x}=\left(M_{RS}+M_{RR}\right)/2$$ (2)

where M_RS_ and M_RR_ are the mortalities of the presumed RS and RR genotypes at dose x, respectively. Chi-square values were then calculated for each concentration as follows:

$$\chi^{2}={\left(F_{1}-pn\right)}^{2}/pqn$$ (3)

where F_1_ is the observed number that had dead in backcross survival bioassays at dose x, n is the number of backcross progeny exposed to dose x, p is the expected mortality, and q = 1 –p. Then the sum of χ^2^ (∑χ^2^) at each concentration was compared with a chi-square distribution with one degree of freedom. The inheritance of resistance is expected to fit the monofactorial model if ∑χ^2^ <χ^2^_0.05_ (df = 1) [30].

## Results

### Selection for Resistance to Cry1F in ACB

A Cry1F-resistant colony of Asian corn borer (ACB-FR) was established through laboratory selection experiments using artificial diet where Cry1F was incorporated. As stated in Meterials and Methods the resistant population was steadily selected with increasing concentrations of Cry1F from 0.05 to 25 μg/g, toxin/diet, for 49 generations. When the susceptibility to Cry1F toxin was assayed in the ACB-BtS and ACB-FR strains, the LC_50_ value was significantly higher in ACB-FR compared with ACB-BtS (Table 1). The differences in LC_50_ values between ACB-BtS and ACB-FR strains and the resulting resistance ratio of more than 1700-fold demonstrates that resistance to Cry1F toxin is achievable for this species.

**Table 1:** Toxicity of 5 Bt toxins against the Asian corn borer strains ACB-BtS and ACB-FR.

Bt toxin	ACB-strains	n^a^	LC_50_ (95%FL) (μg/g)	RR^b^ (95%FL)	Slope±SE	χ^2^	df (χ^2^)
Cry1F	ACB-BtS	672	0.57 (0.36–0.83)	-	2.76 ± 0.36	11.5	10
	ACB-FR	1248	>1000	>1754	nd^c^	nd	22
Cry1Ab	ACB-BtS	672	0.23 (0.14–0.33)	-	2.76 ± 0.40	16.9	10
	ACB-FR	672	5.13 (4.12–6.27)	22.8 (16.9–30.9)	2.51 ± 0.40	5.1	10
Cry1Ac	ACB-BtS	768	0.18 (0.06–0.33)	-	1.11 ± 0.17	12.8	12
	ACB-FR	672	4.88 (3.91–5.97)	26.9(15.7–46.0)	2.44 ± 0.38	4.4	10
Cry1Ah	ACB-BtS	672	0.21 (0.12–0.31)	-	2.56 ± 0.40	15.9	10
	ACB-FR	576	0.78 (0.38–1.34)	3.7 (2.3–5.9)	1.27 ± 0.20	7.7	8
Cry1Ie	ACB-BtS	672	1.69 (0.41–2.46)	-	0.9 ± 0.25	5.6	10
	ACB-FR	576	1.03 (0.78–1.26)	0.6 (0.4–1.0)	0.96 ± 0.13	4.6	8

^a^ n, number of larvae tested.

^b^ RR, resistance ratio

^c^ nd, not determined, indicates that the Probit regression line could not be determined because the range of Cry1F concentrations needed to cause significant response exceed the range tested.

### Cross-Resistance

The LC_50_ values for Cry1Ab and Cry1Ac were significantly higher in the ACB-FR strain compared to the ACB-BtS strain, i.e. selection with Cry1F led to a 22.8-fold and a 26.9-fold decreases of susceptibility to Cry1Ab and Cry1Ac toxins, respectively (Table 1), indicating that the ACB-FR strain is slightly cross-resistant to both toxins. The ACB-FR strain showed marginal cross-resistance to Cry1Ah with a 3.7-fold increase in LC_50_ value, while no cross-resistance was detected with Cry1Ie toxin (Table 1).

### Maternal Effects and Sex Linkage

To determine the mode of inheritance to Cry1F, Cry1Ab and Cry1Ac at lethal concentrations, the sensitivity of F_1_ progenies to these toxins was tested. The LC_50_ for the F_1_ progeny from reciprocal crosses to Cry1F was significantly greater than the LC_50_ for the susceptible parental strain (0.57 μg/g) and significantly less than the LC_50_ for the resistant parental strain (>1000 μg/g see Table 1) with LC_50_ values of 17.69 μg/g and 18.62 μg/g (Tables 1 and 2). These values were not significantly different from each other based on overlap of their fiducial limits indicating that resistance was autosomally inherited with no maternal effects. Analysis of equality and parallel tests showed that the hypothesis of equality (equal slopes, equal intercepts) and the hypothesis of parallelism (equal slopes) were not rejected (P>0.05). These data confirmed that there are no significant differences in bioassays of the reciprocal crosses indicating that inheritance of resistance to Cry1F is autosomal with no maternal effects.

**Table 2:** Response of F_1_ progenies of Asian corn borer to Cry1F, Cry1Ab and Cry1Ac.

Bt toxin	Cross	n^a^	LC_50_ (95%FL) (μg/g)	RR^b^ (95%FL)	Slope±SE	χ^2^	df (χ^2^)
Cry1F	R_♀_×S_♂_	768	17.69 (13.88–26.21)	29.8 (22.7–39.1)	2.66 ± 0.62	8.6	12
	S_♀_×R_♂_	768	18.62 (15.53–21.52)	32.7 (25.0–42.9)	3.47 ± 0.48	5.8	12
Cry1Ab	R_♀_×S_♂_	672	1.34 (0.96–1.80)	6.0 (4.3–8.2)	1.85 ± 0.29	9.4	10
	S_♀_×R_♂_	672	1.20 (0.76–1.62)	5.3 (3.9–7.3)	2.30 ± 0.32	13.5	10
Cry1Ac	R_♀_×S_♂_	672	1.67 (1.18–2.11)	9.2 (5.2–16.1)	1.88 ± 0.42	3.7	10
	S_♀_×R_♂_	672	1.36 (0.79–1.86)	7.5 (4.2–13.3)	1.98 ± 0.34	6.7	10

^a^ n, number of larvae tested.

^b^ RR, resistance ratio

Cry1Ab assays showed LC_50_ values of 1.34 μg/g and 1.20 μg/g that were intermediate in resistance to their respective resistant and susceptible parents, and were not significantly different from one another. Finally, Cry1Ac assays on F_1_ offspring showed LC_50_ values of 1.67 μg/g and 1.36 μg/g, and the LC_50_ values of the F_1_ offspring were not significantly different from each other (Table 2). These observations suggest that the gene(s) for resistance to Cry1Ab and Cry1Ac were primarily autosomal.

### Estimation of the Degree of Dominance

The level of dominance is obtained by the calculation of effective dominance at different toxin concentrations. *h* varied with concentration, from dominant inheritance at low concentrations to recessive inheritance at high concentrations. For example, results showed partially dominance at 0.5 μg/g (*h* = 0.90), and declined to incomplete-recessive by treatment concentrations of 20.0 μg/g (*h* = 0.44) and 50.0 μg/g (*h* = 0.05) (Table 3).

**Table 3:** Effective of dominance (*h*) of resistance to Cry1F in Cry1F-selected Asian corn borer.

Concentration (μg/g)	Strains	Survival (%)	Fitness	*h*
0.5	ACB-BtS	55.2	0.59	
	ACB-FR	93.8	1.00	
	ACB-FRS	89.6	0.96	0.90
5	ACB-BtS	0	0	
	ACB-FR	92.7	1.00	
	ACB-FRS	79.2	0.85	0.85
20	ACB-BtS	0	0	
	ACB-FR	85.4	1.00	
	ACB-FRS	37.5	0.44	0.44
50	ACB-BtS	0	0	
	ACB-FR	84.4	1	
	ACB-FRS	4.2	0.05	0.05

Fitness of the susceptible parent and the reciprocal cross was estimated from the survival rate of the larvae at a specific treatment concentration divided by the survival rate of the resistant parent at the same concentration.

### Number of Loci Influencing the Inheritance

The backcross population was bioassayed with Cry1F and tested for goodness-of-fit to a monofactorial model. The pattern of response was not consistent with a monofactorial model ($\sum \chi^{2}=44.13>\sum \chi_{0.05}^{2}=3.84$ (df = 1)) (Table 4). Thus, these data let us conclude that the resistance to Cry1F toxin in Asian corn borer ACB-FR may be under polygenic control.

**Table 4:** Test of monogenic mode of inheritance of resistance to Cry1F in Cry1F-selected Asian corn borer.

Concentration (μg/g)	Actual mortality (%)	Expected mortality (%)	χ^2^
1	6.3	10.4	1.77
5	15.6	14.1	0.20
10	25.0	21.9	0.56
50	64.6	55.7	3.07
100	89.6	58.4	38.53
∑χ^2^			44.13

## Discussion

The ACB-FR strain achieved more than 1700-fold resistance to Cry1F after 49 generations via chronic exposure to purified Cry1F toxin under moderate selection pressure. Compared with previous reports, many factors may account for the level of resistance, such as the intensity of selection in each generation, the number of generations selected, the source and activation state of the selective agent, the toxin concentration, the difference in proteolytic activation and detoxification among others [31–33].

Selection for Cry1F resistance resulted in slight cross-resistance to Cry1Ab and Cry1Ac with resistance ratios of 22.8- and 26.9-fold, respectively. It has been reported that Cry1F differs from Cry1A toxins in both its spectrum of insecticidal activity against lepidopteran larvae, and its amino acid sequence [34]; Cry1Ab shows 71% identity to Cry1F at the amino acid level, while Cry1Ac shows 69% identity with Cry1F as judged by ClustalW2 analysis (http://www.ebi.ac.uk/Tools/msa/clustalw2/). The moderate-level of cross-resistance among the three toxins suggests that Cry1F, Cry1Ab and Cry1Ac may share at least one receptor on the brush border membrane vesicles (BBMV) that is not very important for Cry1Ab and Cry1Ac toxicity. It was previously shown that Cry1F and Cry1Ab bind to similar 150 and 140 kDa proteins in ACB BBMV [35]. In other lepidopteran species such as *Chloridea virescens*, *Helicoverpa armigera*, *Helicoverpa zea*, *Spodoptera exigua*, *O*. *nubilalis*, and *S*. *frugiperda*, it has been shown that Cry1A toxins share binding sites with Cry1F [10, 36–39]. Furthermore, cross-resistance patterns of Cry1F with other Cry proteins in some lepidopteran insects have also been reported. A Cry1F-selected strain of European corn borer that showed more than 3000-fold resistance was as susceptible to Cry1Ab and Cry9C as the unselected control strain, but showed a low level of cross-resistance (7-fold) to Cry1Ac [40]. In agreement with this finding, binding analyses showed that Cry1F did not compete for binding sites with Cry1Ab or Cry9C on *O*. *nubilalis* midgut BBMV [41]. Selection with Cry1Ab on *O*. *nubilalis* resulted in 51.0-fold resistance to Cry1Ac, whereas low levels (less than 5-fold) of cross-resistance were detected with Cry1F [42]. In the case of *S*. *frugiperda* it was shown that field evolved resistance to Bt-corn expressing Cry1F resulted in low cross-resistance to Cry1Ab and Cry1Ac toxins [9, 10]. In contrast to the cross-resistance to Cry1Ab and Cry1Ac observed in ACB-FR, this strain showed no reduced susceptibility to Cry1Ah and CryIe toxins. Cry1Ah, a novel Cry1A toxin [43], as well as Cry1Ie, exhibited high toxicity against lepidopteran larvae of *O*. *furnacalis* [44]. Lack of cross-resistance of ACB-FR to Cry1Ah and CryIe are likely to be due to the lack of shared binding sites in ACB.

LC_50_ values of the F_1_ progeny from reciprocal crosses between the two parental strains, ACB-BtS and ACB-FR, indicated that resistance to Cry1F was inherited as an autosomal trait with no maternal effects. These results are consistent with earlier findings on other insects, including inheritance of Cry1Ab and Cry1Ac resistance in laboratory-selected Asian corn borer [44], *Culex quinquefasciatus* to Cry11Aa + Cry4A + Cry4B [45], *Helicoverpa armigera* to Cry1Ac and Cry2Ab [46, 47], *O*. *nubilalis* to Dipel ES and Cry1F [48, 49], and *S*. *frugiperda* to Cry1F maize [50]. However, there are few reports where the gender of the resistant parents had an influence on the survival of the F_1_ hybrid progeny. Leaf dip bioassays suggested that resistance to Cry1Ac had some maternal influence in *P*. *xylostella* collected from Malaysia [7]. A maternal influence associated with inheritance of Cry1Ab resistance in a field-derived strain of *O*. *nubilalis* was also identified after analysis of concentration–mortality curves of reciprocal crosses [8]. In other studies, resistance to Dipel ES in a laboratory-selected population of *O*. *nubilalis* and resistance to the 130-kDa δ-endotoxin protein in *C*. *virescens* was found to be incompletely dominant [48, 51]. These results demonstrate that knowledge of the inheritance of resistance in different species is required to devise appropriate resistance management strategies.

The effective dominance *h* varied from 0.05 to 0.90, indicating that dominance was dependent on toxin concentration, with resistance being nearly recessive at a high concentration. However, the dominance increased as the concentration decreased. Similar findings have been reported in other species [7, 8, 33, 52]. These results indicate that a high dose of Cry1F in transgenic corn is necessary for a refuge strategy to be successful in delaying resistance. For some species, a high level of variability in the degree of dominance has been observed in different populations, where a strong example may be found in diamondback moth collected from Hawaii and South Carolina [52]. Greenhouse tests indicated that dominance may vary depending on different levels of Bt toxin expression in tissues during different plant stages (e.g. vegetative-stage and reproductive-stage) [8]. In Brazil, the resistance of *S*. *frugiperda* to Cry1F-maize is incompletely recessive and Bt-maize is not high dose for *S*. *frugiperda* [53]. These studies of inheritance of resistance are based on the general assumption that the parent populations are homozygous. In previous studies, variation in the resistance of progeny of F_1_ hybrid crosses showed that resistant alleles were present in susceptible laboratory populations and that susceptible alleles were present in selected resistant populations [28, 54, 55]. For some insects, the recessivity increased with higher levels of resistance in the selected strains [31].

The method used in the present study to determine the number of loci involved in resistance is based on the expected mortality of offspring from the RS × RR backcross at each toxin dose. The test of monogenic mode did not fit the data, revealing that the resistance is polygenic in lab-selected strain ACB-FR. The null hypothesis tested in the standard backcross method is that resistance is controlled by one locus with two alleles [30]. If the resistance had been controlled entirely by one locus with two alleles, the sole RR allele would have been fixed in several generations of selection and further increase in resistance would not have occurred [56]. The number of genes contributing to resistance appears to be different in different insect species and may even differ for individual toxins and under different selection regimes (weaker selection can allow polygenic weak resistance mechanisms to develop through multiple small increases in fitness). Analysis of survival and growth of progeny from backcrosses suggest that resistance to Cry1Ac in a field-derived strain of pink bollworm was controlled primarily by one or a few major loci [56]. Backcrossing studies indicated that resistance to Cry1Ab toxin was polygenic in Cry1Ab-selected Asian corn borer (ACB-AbR), but monogenic in Cry1Ac-selected Asian corn borer (ACB-AcR), while resistance to Cry1Ac toxin corresponds to monogenic inheritance in both ACB-AbR and ACB-AcR colonies [44]. In a Cry1F-selected population of European corn borer, a single locus or a set of tightly linked loci, is responsible for resistance [49], and Cry1Ab resistance was determined to be polygenic in a field-derived *O*. *nubilalis* strain [8]. In some cases, the resistance was primarily monogenic, but polygenic as resistance increased [31].

In summary, laboratory-selected Asian corn borer have developed a high level of resistance to Cry1F but with limited cross-resistance to other toxins, and provided an opportunity to determine the inheritance of Cry1F resistance in this strain. The results presented here could have significant implications for resistance management strategies,especially for the high-dose/ refuge strategy, which functions effectively with recessive traits. These results also indicated that the presence of Cry1F and Cry1Ah or Cry1Ie may significantly slow the development of resistance. In the future, greenhouse experiments with Cry1F-expressing corn hybrids could be carried out to investigate the existence of fitness costs associated with resistance.
